# Hyper-Branched Gold Nanoconstructs for Photoacoustic Imaging in the Near-Infrared Optical Window

**DOI:** 10.1021/acs.nanolett.3c02177

**Published:** 2023-10-05

**Authors:** Myeongsoo Kim, Don VanderLaan, Jeungyoon Lee, Ayoung Choe, Kelsey P. Kubelick, Jinhwan Kim, Stanislav Y. Emelianov

**Affiliations:** † Petit Institute for Bioengineering and Bioscience, Georgia Institute of Technology, Atlanta, Georgia 30332, United States; ‡ Wallace H. Coulter Department of Biomedical Engineering, Georgia Institute of Technology and Emory University School of Medicine, Atlanta, Georgia 30332, United States; § School of Electrical and Computer Engineering, Georgia Institute of Technology, Atlanta, Georgia 30332, United States

**Keywords:** photoacoustic imaging, hyper-branched gold nanoconstructs, exogenous contrast agents, blackbody absorption, cancer imaging

## Abstract

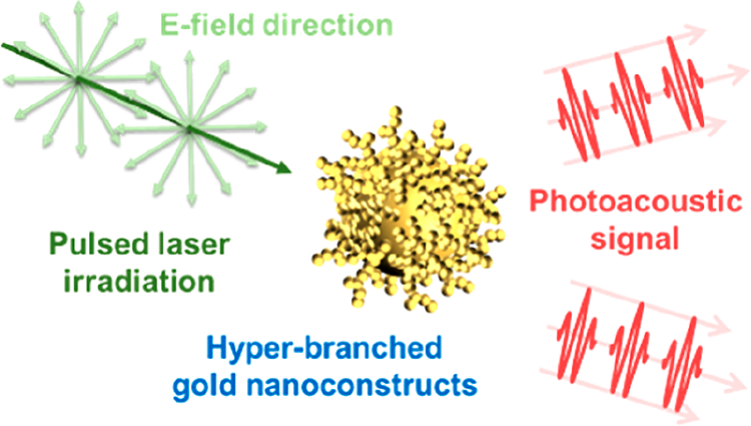

In plasmonic nanoconstructs (NCs), fine-tuning interparticle interactions at the subnanoscale offer enhanced electromagnetic and thermal responses in the near-infrared (NIR) wavelength range. Due to tunable electromagnetic and thermal characteristics, NCs can be excellent photoacoustic (PA) imaging contrast agents. However, engineering plasmonic NCs that maximize light absorption efficiency across multiple polarization directions, i.e., exhibiting blackbody absorption behavior, remains challenging. Herein, we present the synthesis, computational simulation, and characterization of hyper-branched gold nanoconstructs (HBGNCs) as a highly efficient PA contrast agent. HBGNCs exhibit remarkable optical properties, including strong NIR absorption, high absorption efficiency across various polarization angles, and superior photostability compared to conventional standard plasmonic NC-based contrast agents such as gold nanorods and gold nanostars. *In vitro* and *in vivo* experiments confirm the suitability of HBGNCs for cancer imaging, showcasing their potential as reliable PA contrast agents and addressing the need for enhanced imaging contrast and stability in bioimaging applications.

Photoacoustic (PA) imaging is a technique that merges the advantages of ultrasound (US) and optical imaging by the conversion of light into thermoacoustic waves upon irradiation of optical absorbers with high-intensity laser pulses.^1−6^ In PA imaging, both endogenous and exogenous contrast agents are employed. Endogenous molecules, such as hemoglobin, are commonly used for the imaging of vasculature associated with hemodynamics and oxygenation.^7,8^ Exogenous contrast agents with nanoscale diameter (<100 nm) and molecular targeting moieties are better suited for imaging cellular and molecular properties of tissue.^9−12^ Particularly in the development of exogenous contrast agents for *in vivo* applications, it is crucial to minimize the background PA signals arising from endogenous molecules.^13−16^ This can be accomplished by designing blackbody-like PA contrast agents that can strongly absorb incident laser pulses within the NIR optical window (650–1300 nm) across multiple light polarization directions. Furthermore, it is crucial for exogenous PA contrast agents to maintain unaltered optical absorption characteristics during successive pulsed laser irradiation, ensuring reliable, high-contrast PA imaging over multiple imaging sessions.

Gold nanoconstructs (GNCs) have emerged as a highly promising class of exogenous PA imaging contrast agents due to their large optical absorption cross sections, shape-dependent tunable optical properties, high heat conductivity, surface functionality, and biocompatibility.^17−23^ For example, anisotropic structures, such as gold nanorods (GNRs) and gold nanoplates, have been widely utilized as PA contrast agents due to their high optical absorption in the NIR optical window.^13,24−26^ However, structural anisotropy of the GNCs leads to the polarization dependence on light absorption.^27^ This polarization dependence results in strong PA signal generation only if the orientation of anisotropic GNCs is parallel to the polarization direction of incident laser light.^28,29^ Furthermore, the anisotropic nature of the GNCs makes these prone to shape transition into spheres upon pulsed laser illumination, resulting in optical absorption changes.^30,31^ Consequently, PA signal decays, especially in the NIR window, rendering anisotropic GNCs unsuitable for continuous PA imaging with high imaging contrast.^24,32,33^ Therefore, for PA imaging using exogenous plasmonic GNCs as an imaging contrast agent, it is critical to devise GNCs with strong optical absorption across multiple polarization angles and high photostability to withstand irradiation with multiple laser pulses.

As an alternative to anisotropic GNCs, leveraging the plasmon coupling of isotropic gold nanospheres (GNSs) provides an effective method for generating optical responses at NIR wavelengths.^34−36^ If two or more GNSs are in close proximity, typically within a few nanometers, their plasmon modes hybridize, leading to a redshift of the optical absorption peak toward longer wavelengths.^34,35,37,38^ In plasmon coupling, the absorption intensity depends on the total number and interparticle distance of proximate GNSs.^34,37^ Therefore, the fabrication of a nanoscale superstructure that comprises (1) closely spaced GNS assemblies to induce intense plasmon coupling and (2) an unpolarized orientation of the GNS assembly, where the GNSs densely branch out in multiple directions, can lead to blackbody-like absorption behavior at NIR frequencies across multiple light polarization directions, resulting in strong PA signals and contrast. While several previous studies have demonstrated the synthesis of blackbody-like GNCs via self-assembly of GNSs for PA imaging applications, this approach still needs improvement in controlling the reproducibility of structural parameters of the GNS assembly, including size uniformity and interparticle distances.^36,39,40^

To create optimal GNS assemblies with dense, outward extending branches, referred to as “hyper-branched gold nanoconstructs” (HBGNCs), it is necessary to precisely control structural parameters of GNSs at the subnanometer scale and to have a comprehensive understanding of optical responses stemming from plasmon coupling. In this study, we introduce a seed-mediated, surface blocker-aided growth approach to synthesize hyper-branched GNS superstructures on gold seed particles, creating HBGNCs. To investigate the optical responses of the designed HBGNCs, such as the optical absorption efficiency and dependence of optical absorption on light polarization, we employ a finite-difference time-domain (FDTD) simulation. Our simulation results indicate that HBGNCs exhibited strong NIR-light absorption with negligible optical scattering across multiple polarization angles. Due to their exceptional optical characteristics, imaging experiments showed that HBGNCs exhibited a stronger PA response compared to traditional GNC-based contrast agents, such as GNRs and gold nanostars (GNSTs). Furthermore, HBGNCs had superior photostability compared to traditional GNCs. Due to the robust PA response of HBGNCs, the PA imaging signal was detectable even at picomolar (pM) concentration ranges. Lastly, we demonstrated the capability of HBGNCs for *in vitro* and *in vivo* PA imaging of cancer cells.

To synthesize HBGNCs with close branch proximity, we carried out a surface-blocker-assisted, seed-mediated growth of 35-nm-sized GNSs (Figure S1), which was developed based on methods used for controlling growth pathways through the interface engineering of underlying seed particles.^41−44^ In this growth process, a blend of silver and halide ions was utilized to form a silver halide complex as a surface blocker to partially passivate the surface of gold seeds.^41,42,44^ Thus, island growth on the gold seeds was promoted to generate a core-island structure, not the layer-by-layer growth that creates a shell-like smooth structure. To further change the growth mechanism from island growth to hyper-branch growth that involves the branching out of GNSs in multiple directions, continual island growth on the previously created islands was promoted by partially blocking the island surface to support branch formation (Figure 1a).

**Figure 1:**
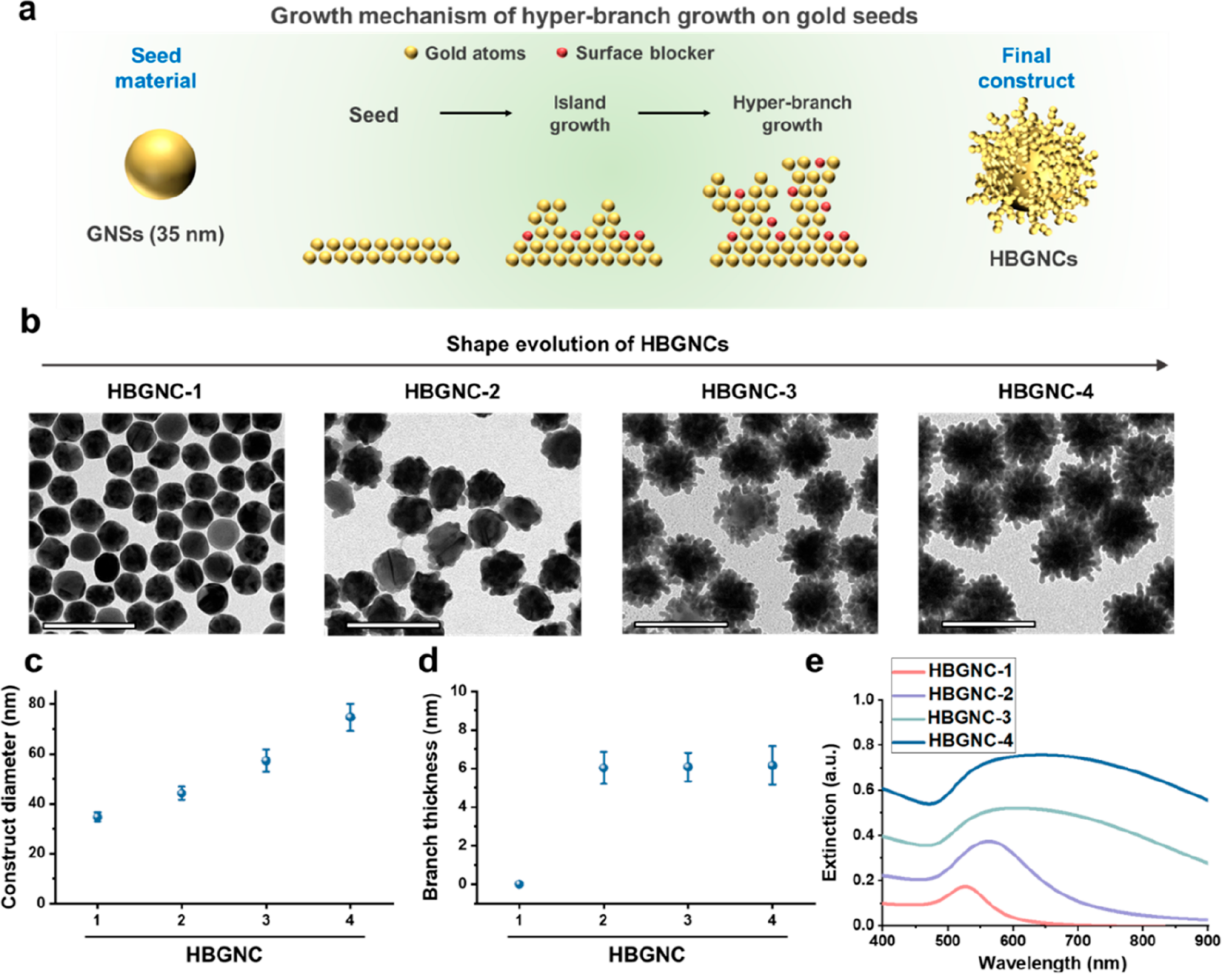
Strategy for the design of HBGNCs via interface engineering during the seed-mediated growth process. (a) Schematic of the seed-mediated growth for the creation of HBGNCs. (b) TEM images of HBGNCs synthesized by modulating the ratio of the number of gold seeds in the growth step against the amounts of gold ion precursors and surface blockers. The scale bars are 100 nm. (c, d) Quantifications of the diameters and branch thicknesses of HBGNCs (*n* = 40). (e) UV–vis-NIR spectra of HBGNCs at 20 pM concentration. Data represent the mean ± standard deviation.

The shape evolution of HBGNCs was investigated by adjusting the number of gold seeds in the growth process against the amounts of gold ion precursors and surface blockers. Based on the volume ratio of the gold ion precursor and gold seed solutions from 0:1 to 0.5:1, four distinct types of HBGNCs were synthesized: HBGNC-1 (no gold ion precursors, i.e., core GNS only), HBGNC-2 (low precursor concentration; the ratio of 0.1:1), HBGNC-3 (medium precursor concentration; the ratio of 0.25:1), and HBGNC-4 (high precursor concentration; the ratio of 0.5:1). The morphology of each construct was observed via transmission electron microscopy (TEM). As the ratio of gold ions to gold seeds in the growth process increased, we noted an increase in the construct diameter from 35 to 75 nm, while maintaining nearly constant branch thickness (Figure 1b–d). Moreover, as the construct diameter and branch proximity of HBGNCs increased (from HBGNC-1 to HBGNC-4), there was a corresponding rise in extinction at NIR frequencies, as measured by UV–vis-NIR spectroscopy (Figure 1e). The increase in optical extinction is attributed to the enhanced interbranch plasmon coupling.

Given that the silver halide complex passivates the surface of gold seeds, subsequently leading to changes in the growth pathway (Figure 1), we conducted further investigations to determine whether adjusting the quantity of the surface blocker on the seed particle could modulate the branch density and interbranch spacing in the HBGNC. To explore the effect of the surface blocker quantity on hyper-branch growth, we modulated the ratio of silver to gold ions in the growth process, while the silver-to-halide ion ratio and the total amount of gold ions were both held constant. Results showed that increasing the silver-to-gold ion ratio from 0.1:5 to 1:5 resulted in a decrease in branch thickness and an increase in branch density. Within the growth process, the construct morphology remained uniform with structural homogeneity up to a silver-to-gold ion ratio of 1:5. Specifically, ratios of 0.5:5 and 1:5 induced hyper-branch growth on the GNS seeds. Conversely, insufficient amounts of surface blockers (ratios of 0.1:5 and 0.25:5) led to island growth without the formation of hyper-branches (Figure S2a). If the silver-to-gold ion ratio exceeded 1:5, we observed the occurrence of free particle nucleation and the formation of nonuniform HBGNCs (Figure S2a). Moreover, silver-to-gold ion ratios of 5:5 and 10:5 resulted in thick branch or shell growth without hyper-branch formation. This observation suggests that excessive amounts of surface blockers in the growth process fully passivated the GNS surface, thus inhibiting hyper-branch growth (Figure S2b). Based on the TEM results demonstrating that the silver-to-gold ion ratios ranging from 0.1:5 to 1:5 ensured HBGNCs to exhibit structural homogeneity with no generation of free nucleated particles, we characterized optical properties of HBGNCs fabricated at the ratios ranging from 0.1:5 to 1:5 via UV–vis-NIR spectroscopy. This result showed that HBGNCs fabricated at the ratio of 1:5 exhibited stronger optical absorption at NIR frequencies due to more efficient interbranch plasmon coupling in close proximity compared to other HBGNCs (Figure S2c). Therefore, we selected a silver-to-gold ion ratio of 1:5 in the seeded growth process to produce HBGNCs with a maximized branch density and structural homogeneity. Low-magnification TEM and scanning electron microscopy (SEM) images confirmed the structural homogeneity of the HBGNCs synthesized at the silver-to-gold ion ratio of 1:5 in the growth process HBGNC-4 (Figures S3 and S4). Results collectively demonstrate that our surface blocker-assisted growth approach enables the synthesis of uniform HBGNCs that exhibit a strong and broad optical response at NIR frequencies.

If HBGNCs are irradiated by laser pulses, PA signal is generated through the conversion of pulsed laser energy into heat pulses and subsequent production of acoustic transients.^13,45,46^ Therefore, an FDTD numerical simulation was carried out to calculate the fraction of absorption and extinction, i.e., absorption efficiency, for HBGNCs within the 700–900 nm spectral range. The simulation model was designed with the structural parameters of HBGNC-4, including the diameter of the core sphere, branch thickness, and branch length, as determined by TEM analysis (Figure 1b–d). Results showed that HBGNCs exhibit strong absorption with a high absorption efficiency of approximately 90% across the NIR spectral region, while GNS seed particles exhibit negligible absorption cross sections (Figure 2a–c). The local electric-field (E-field) enhancement was observed in the gap region between closely spaced branches, suggesting that the coupling and hybridization of plasmon modes between proximate branches lead to large absorption cross sections across NIR wavelengths (Figure 2d). We further analyzed the polarization dependence of HBGNCs on optical absorption by estimating their maximum absorption cross sections at varied polarization angles from 0° to 90° (Figure 2e). The HBGNCs display large absorption cross sections across all polarization angles. Together, results demonstrate the capability of HBGNCs to absorb laser pulses with exceptional absorption efficiency at various light polarization directions, leading to strong PA signal generation with high imaging contrast.

**Figure 2:**
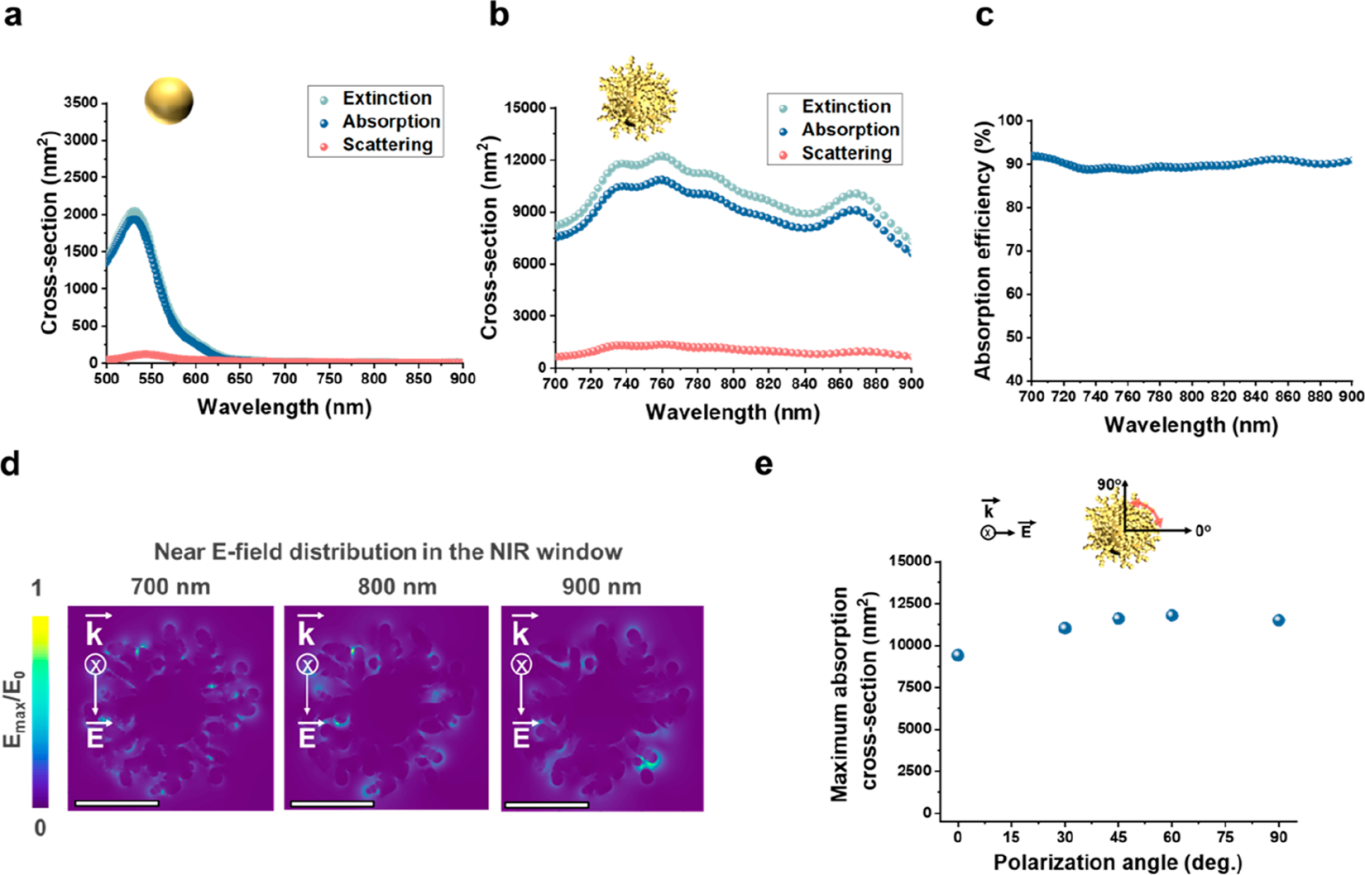
Theoretical analysis of the optical properties of HBGNCs in NIR wavelength range. (a, b) Calculated optical extinction, absorption, and scattering cross sections of GNS seeds and HBGNCs (HBGNC-4). (c) Estimated absorption efficiency of HBGNCs within the 700–900 nm spectral range. (d) The near electric-field distribution in the HBGNCs at 700, 800, and 900 nm, respectively. The scale bars are 40 nm. (e) Calculated maximum absorption cross sections of HBGNCs at different polarization angles. In simulations, a single HBGNC was dispersed in water and excited by linearly polarized light at various polarization angles.

PA signal generation from HBGNCs (HBGNC-4) within the NIR wavelength range (700 to 900 nm) was tested using a polyethylene tube phantom (Figure 3a). The HBGNCs exhibited remarkable PA amplitude within this spectral region due to their strong optical absorption in the NIR range, compared to GNS seed particles (Figure 3b). The PA signal amplitude from HBGNCs was discernible even at an HBGNC concentration of 26 pM with a clear linear relationship (*R*^2^ = 0.99) between PA signal amplitude and HBGNC concentration (Figure 3c).

**Figure 3:**
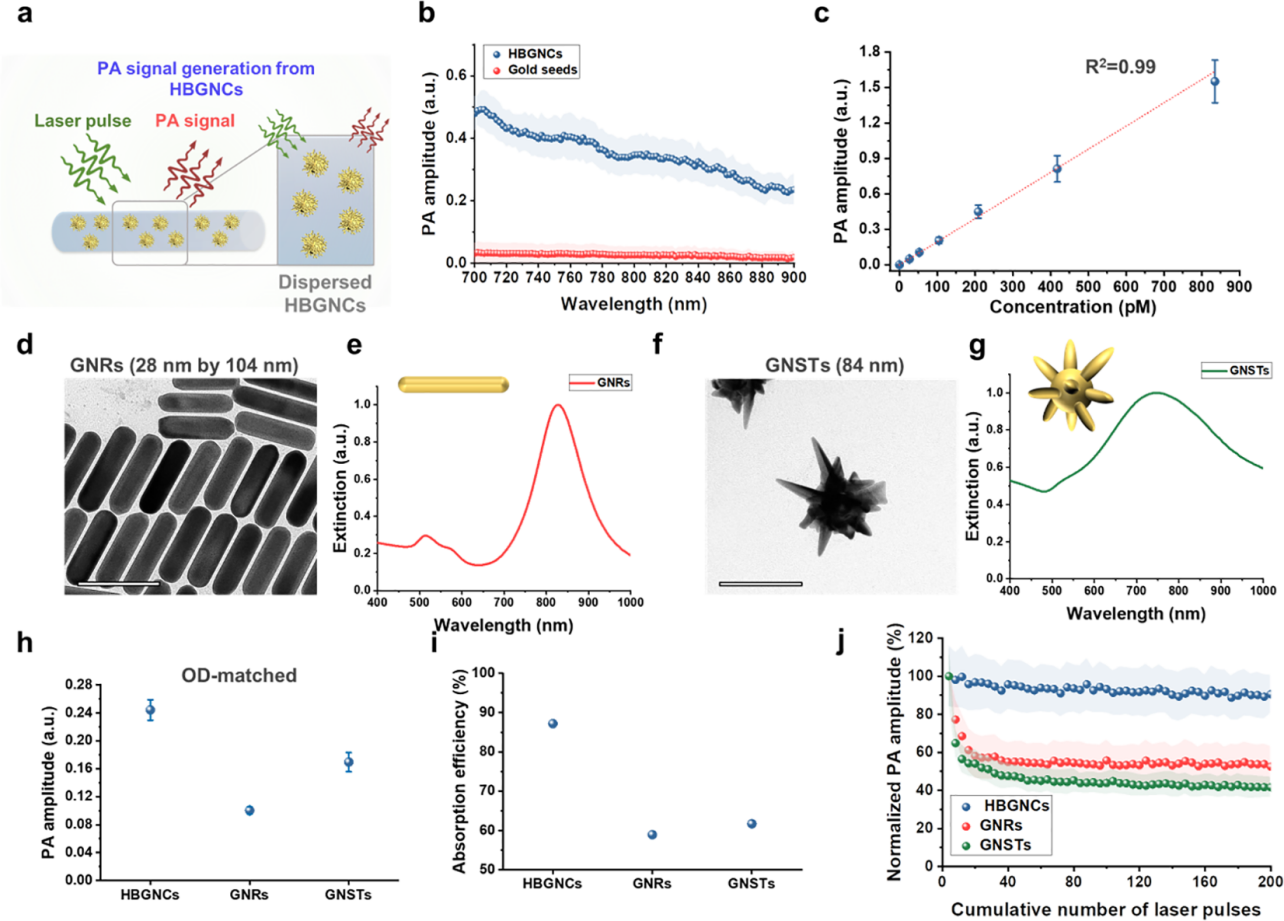
Analysis of PA signal generation from HBGNCs. (a) Schematic of PA experiment to investigate PA signal generation from HBGNCs (HBGNC-4). (b) PA signal generation from HBGNCs and GNS seeds at a particle concentration of 200 pM (*n* = 5). (c) PA signal generation from HBGNCs at different concentrations under 700 nm wavelength pulsed laser illumination. (d–g) TEM images and UV–vis-NIR spectra of GNRs and GNSTs. The scale bars are 100 nm. (h) PA signal generation from OD-matched GNCs with different morphologies, including hyper-branch, rod, and star. PA signals from the HBGNCs, GNRs, and GNSTs were acquired at wavelengths of 700, 830, and 750 nm, respectively (*n* = 5). (i) Calculated optical absorption efficiency of the HBGNCs, GNRs, and GNSTs, at 700, 830, and 750 nm wavelengths, respectively. (j) PA signal generation from the different GNCs under pulsed laser illumination at a fluence of 10 mJ cm^–2^ (*n* = 4). Data represent the mean ± standard deviation.

A comparative analysis of PA signal generation from HBGNCs and conventional plasmonic PA contrast agents, including GNRs and GNSTs, was performed (Figures 3d–g and S5). All of the GNCs had similar dimensions. In the 700–900 nm spectral region, the maximum optical absorption wavelength for HBGNCs, GNRs, and GNSTs was found to be 700, 830, and 750 nm, respectively. PA responses from the water-based solutions (optical density, OD = 3) of the different GNCs at their corresponding maximum absorption wavelength were measured using laser pulses with a laser fluence of 10 mJ cm^–2^ and linear light polarization parallel to the tube. Results demonstrated that PA signal generation from HBGNCs was 2.43-fold and 1.44-fold higher than that from GNRs and GNSTs, respectively (Figure 3h). Furthermore, to assure the PA signal differences were not attributed to different interfacial heat resistances induced by different surface ligands, all GNCs were then capped with the same polyethylene glycol (PEG) ligands while maintaining constant OD. Results showed that the PA signal amplitude of HBGNCs was 2.17-fold and 1.62-fold higher than that of GNRs and GNSTs, respectively (Figure S6a). Moreover, the different GNCs displayed comparable PA amplitudes before and after PEGylation, showing no significant difference (Figure S6b–d). The results indicate that the PEGylation process had a negligible influence on PA signal generation from the different GNCs.

FDTD simulation results indicated that the absorption efficiency of HBGNCs is significantly higher than that of GNRs (∼65%) and GNSTs (∼55%) at their peak optical absorption wavelengths (Figures 3i and S7). Furthermore, as discussed previously (Figure 2e), HBGNCs displayed strong absorption across all light polarization directions, while anisotropic structures such as GNRs showed a significant drop in absorption if their orientation was perpendicular to the light polarization direction (Figure S7). The polarization dependence of GNR’s optical absorption leads to strong PA signal generation only if GNRs are parallel to the laser polarization direction,^29^ which means that GNRs orthogonally orientated to the laser light polarization direction generate poor PA signal generation due to the low absorption cross-section of GNRs (Figure S7c). Thus, the higher PA response from HBGNCs can be explained by the superior absorption efficiency and polarization-angle-independent absorption of HBGNCs. Furthermore, the higher surface-to-volume ratio of HBGNCs compared to GNRs and GNSTs could facilitate more efficient heat transfer into the surrounding medium, thereby enhancing PA signal generation.^13,28,46,47^

In PA imaging, the photostability of GNCs is critical, especially if imaging is performed over multiple imaging sessions. Therefore, the photostability of HBGNCs was tested in comparison to the GNRs and GNSTs under pulsed laser illumination for 200 pulses at 10 mJ cm^–2^. The laser excitation wavelengths corresponded to the peak optical absorption wavelength of each contrast agent, i.e., 700, 830, and 750 nm for HBGNCs, GNRs, and GNSTs, respectively. While GNRs and GNSTs exhibit dramatic PA signal decay within 200 pulses, HBGNCs produce a consistent and stable PA signal amplitude without any signal decay (Figure 3j). The poor photostability of GNRs and GNSTs could be explained by the reshaping behavior of anisotropic GNCs below their melting temperature.^30^ In addition, localized E-field enhancement at the tips of anisotropic GNCs results in high local temperatures at the construct tips.^48,49^ (Figure S8). The high-temperature localization can cause the construct tips to melt more easily than the body of the construct,^48,49^ leading to the morphological transition of the anisotropic shape into spheres via atomic diffusion,^30,50^ followed by decreased optical absorption and subsequent PA signal decay. In contrast, small GNSs that were used in HBGNC synthesis have been shown to exhibit a melting temperature comparable to that of bulk gold^51^ and a high photodamage threshold of approximately 50 mJ cm^–2^ under pulsed laser illumination.^52^ In addition, HBGNCs are likely to generate heat uniformly throughout the entire construct body, as evidenced by the evenly distributed near E-field distribution (Figure 2d). The HBGNCs exhibited consistent PA signal generation and maintained their morphological characteristics up to a laser fluence of 15 mJ cm^–2^ (Figure S9). Together, the findings showcase the potential of our HBGNCs to surpass conventional plasmonic contrast agents, in providing reliable and high-contrast PA imaging across multiple imaging sessions.

We proceeded to demonstrate the potential of HBGNCs for PA imaging of cancer by incorporating a molecular targeting moiety. To facilitate cellular interactions and internalization by cancer cells, the surface of HBGNCs was functionalized with cyclic arginine-glycine-aspartic acid (cRGD) peptides, which selectively bind to integrin α_v_β_3_, a receptor overexpressed in various cancer cell types^53,54^ (Figure 4a). Zeta potential analysis showed serial changes in the surface charge of HBGNCs in each process of the cRGD functionalization, validating the successful cRGD functionalization on HBGNCs (Figure S10). The density of cRGD ligands was quantified as 6,278 ± 1,456 ligands per HBGNC (Figure S11). Next, we performed *in vitro* US/PA imaging on MDA-MB-231 breast cancer cells incubated with cRGD-functionalized HBGNCs (cRGD-HBGNCs) at a concentration of 0.075 nM, as guided by the toxicity evaluation of HBGNCs (Figure S12). The HBGNC-labeled cells were then embedded into a tissue-mimicking phantom and underwent US/PA imaging at 700 nm wavelength (Figure 4b). The HBGNC-labeled cells presented a distinct PA signal and high contrast in comparison to unlabeled cells (Figure 4c), with the detection limit extending to a cell concentration as low as ∼20 cells/μL (Figure S13). Furthermore, MDA-MB 231 cancer cells labeled with cRGD-HBGNCs exhibited an approximately 70% higher PA signal amplitude compared with cells labeled with PEGylated HBGNCs or cyclic RAD (scrambled RGD)-modified HBGNCs (Figure S14). This significant increase in PA signal amplitude suggests that the coupling of RGD moieties to HBGNCs can enhance the target specificity of HBGNCs toward cancer cells. This enhancement is attributed to the cellular internalization of the HBGNCs facilitated by the recognition of integrin α_v_β_3_ receptors on cancer cells.

**Figure 4:**
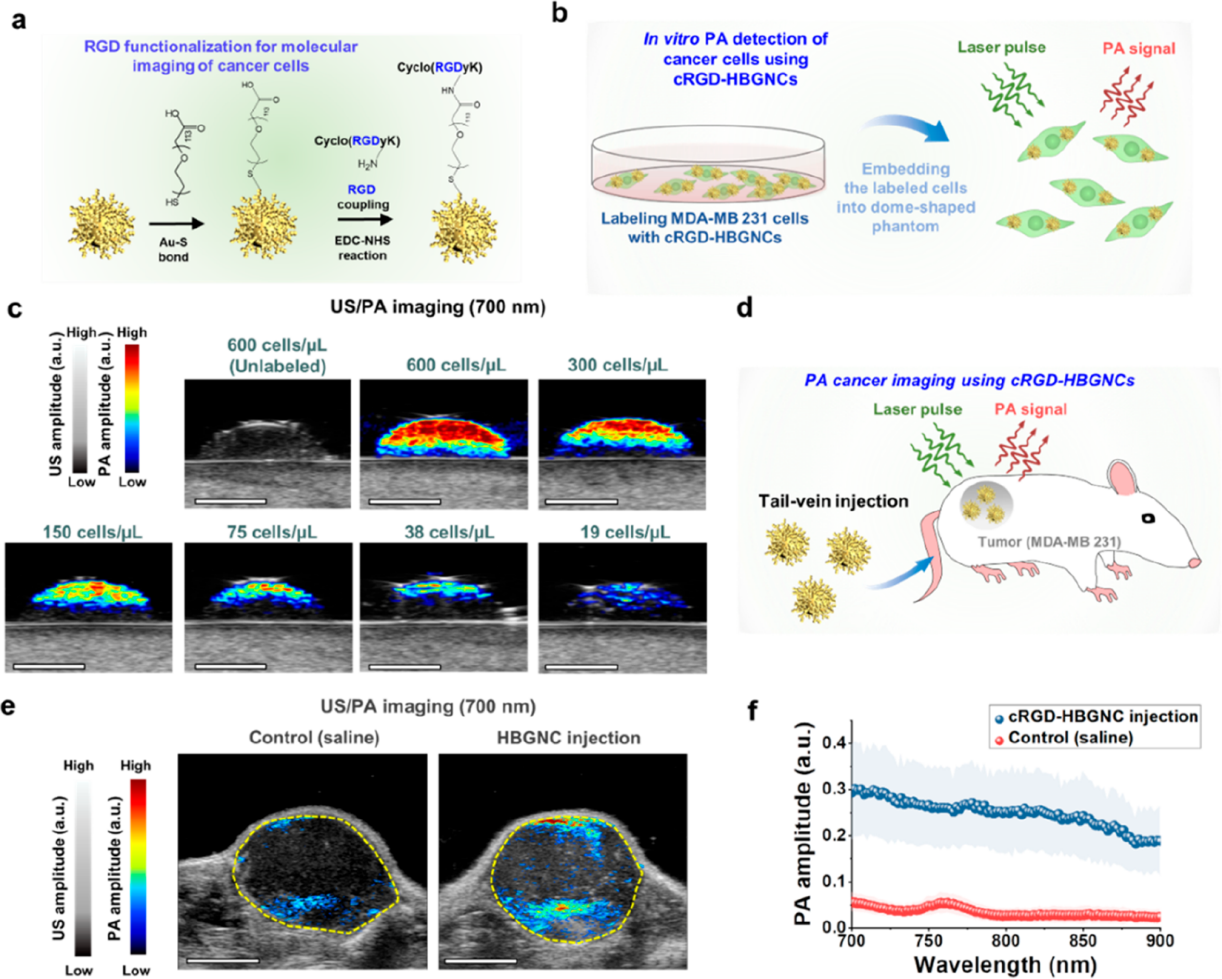
*In vitro* and *in vivo* PA imaging using HBGNCs. (a) Schematic of surface functionalization process for coupling cyclic RGD (cRGD) ligands to HBGNCs. (b) Schematic of *in vitro* US/PA imaging by utilizing a dome-shaped tissue-mimicking phantom containing cancer cells labeled with cRGD-HBGNCs. (c) US/PA images of the dome phantoms containing labeled MDA-MB 231 cells at different cell concentrations under 700 nm pulsed laser illumination. The scale bars are 4 mm. (d) Schematic of *in vivo* PA cancer imaging using the cRGD-HBGNCs. (e) US/PA images of the tumor region of a mouse intravenously injected with saline (control) or cRGD-HBGNCs. The scale bars are 4 mm. The yellow dotted contour indicates the tumor region based on US imaging. (f) Corresponding PA spectra of the tumor site (*n* = 3). Data presented as the mean ± standard deviation. The imaging experiments were repeated independently three times and similar results were received.

Lastly, we demonstrated the *in vivo* PA imaging capability of HBGNCs as an efficient and stable imaging contrast agent. Preliminarily, to confirm the *in vivo* imaging potential and photostability of HBGNCs, HBGNCs, suspended in Matrigel, were administered subcutaneously in healthy mice, followed by US/PA imaging (Figure S15). While the Matrigel only (control) did not generate any noticeable PA signal *in vivo*, mice that received Matrigel with HBGNCs exhibited a distinct PA signal and imaging contrast within the 700–900 nm spectral region, maintaining consistent PA amplitude for 1,000 pulses at 700 nm (Figure S15), owing to HBGNC photostability as demonstrated earlier (Figure 3j). Then, we demonstrated cRGD-HBGNCs for PA cancer detection using systemic delivery of cRGD-HBGNCs in MDA-MB-231 tumor-bearing xenograft mice (Figure 4d). Tumor regions underwent US/PA imaging 24 h following intravenous injection of cRGD-HBGNCs or saline (control). The ultrasound (US) images for both the saline control and HBGNC-injected mice show excellent anatomical detail, and an increased PA imaging signal at 700 nm was observed solely in the mice that received cRGD-HBGNCs (Figures 4e and S16a). Quantitative analysis revealed that the PA signal amplitude from the tumor region in the 700–900 nm spectral region in the HBGNC-injected group was approximately 7-fold higher than that in the saline-injected group (Figures 4f and S16b). Collectively, these results confirmed the feasibility of the HBGNCs as potent exogenous contrast agents for *in vivo* US/PA imaging.

In summary, we have successfully fabricated HBGNCs as PA contrast agents that exhibit light-polarization-independent blackbody-like optical absorption within the NIR optical window. Our experimental and theoretical characterizations for the optical properties of HBGNCs demonstrate strong PA signal and imaging contrast. The strong PA response from our HBGNCs results in an exceptionally low detection limit of 26 pM. Moreover, our HBGNCs exhibit a higher photodamage threshold than traditional plasmonic PA contrast agents, such as GNRs and GNSTs, underscoring the advantages of HBGNCs for reliable, high-contrast PA imaging over multiple imaging sessions. *In vivo* US/PA imaging using cRGD-HBGNCs as a targeted contrast agent showcased the capability to monitor the tumor region with high PA imaging contrast.

Compared to traditional plasmonic contrast agents with anisotropic morphologies that are widely accepted as standard *in vivo* PA imaging agents, our HBGNCs present multiple advantages for PA imaging. First, HBGNCs can absorb incident laser pulses from multiple angles and light polarization directions, leading to strong PA signal generation irrespective of their orientation. Second, HBGNCs exhibit a high absorption efficiency of approximately 90% to efficiently generate the PA signal by minimizing local fluence reduction due to optical scattering. Third, HBGNCs can transfer heat pulses into the surrounding medium more efficiently than other contrast agents due to the higher surface-to-volume ratio of HBGNCs. This superior heat transfer results in higher PA signal and contrast. Lastly, HBGNCs are less susceptible to photodamage and possess a higher photodamage threshold than traditional plasmonic contrast agents absorbing light in the NIR wavelength range, such as GNRs and GNSTs. This superior photostability suggests the potential of our HBGNCs to outperform traditional plasmonic imaging agents for high-contrast, longitudinal PA imaging. Despite the numerous advantages of our HBGNCs for PA imaging, their blackbody-like optical characteristic may pose some challenges in image post-processing, particularly when it comes to spectral unmixing with endogenous optical absorbers, such as melanin. Nevertheless, our strategy to create HBGNCs with blackbody characteristics will have a significant impact on the design of plasmonic NC-based PA contrast agents that show high contrast across multiple imaging sessions in the NIR window for *in vivo* PA imaging applications.

## Supporting Information Available

Experimental details and supplementary figures, including preparation and characterization of HBGNCs, calculated near E-field distributions of GNCs with different morphologies, cell viability tests, quantitative analysis of PA signal generation from cancer cells labeled with HBGNCs, and *in vivo* PA signal generation from HBGNCs; Figures S1–17 (PDF)
