# Supplementary material for: Hyper-Branched Gold Nanoconstructs for Photoacoustic Imaging in the Near-Infrared Optical Window
Source: Nano Lett. 2023 Oct 5;23(20):9257–65. doi: 10.1021/acs.nanolett.3c02177 (PMC10603794; doi:10.1021/acs.nanolett.3c02177)
Supplement: Supplementary file 1 — nl3c02177_si_001.pdf [file nl3c02177_si_001.pdf]

Supplementary Information

Hyper-branched gold nanoconstructs for photoacoustic imaging in the near-infrared optical window

Myeongsoo Kim[a,b], *Don VanderLaan*[c], *Jeungyoon Lee*[c], *Ayoung Choe*[b,c], *Kelsey P. Kubelick*[b,c], *Jinhwan Kim*[b,c,d*], *Stanislav Y. Emelianov*[a,b,c,*]

[a] Petit Institute for Bioengineering and Bioscience, Georgia Institute of Technology, Atlanta, GA, 30332, US

[b] Wallace H. Coulter Department of Biomedical Engineering, Georgia Institute of Technology and Emory University School of Medicine, Atlanta, GA 30332, USA

[c] School of Electrical and Computer Engineering, Georgia Institute of Technology, Atlanta, GA, 30332, USA

[d] The current affiliation of the author is the Department of Surgery, School of Medicine, University of California Davis, Sacramento, CA 95817, USA and the Department of Biomedical Engineering, University of California Davis, Davis, CA 95616, USA.

[*] Corresponding Authors: Stanislav. Y. Emelianov (stas@gatech.edu) and Jinhwan Kim (jjnkim@ucdavis.edu)

Supplementary Note 1. Experimental Details

1.1. Materials

Gold (III) chloride trihydrate ($HAuCl_4 \cdot 3H_2O$), sodium borohydride ($NaBH_4$), sodium citrate tribasic dihydrate (Na_3Cit), potassium iodide (KI), L-ascorbic acid (LAA), polyvinylpyrrolidone (PVP, molecular weight 55k), silver nitrate ($AgNO_3$), and dimethyl sulfoxide (DMSO), were purchased from Sigma Aldrich (St. Louis, MO, USA). N-Hydroxysulfosuccinimide Sodium Salt (Sulfo-NHS) was purchased from bioWORLD (Dublin, OH, USA) and 1-(3-Dimethylaminopropyl)-3-ethylcarbodiimide (EDC) was purchased from Alfa Aesar (Haverhill, MA). Carboxymethyl-PEG-thiol (CM-PEG-SH, molecular weight 5k) was purchased from Laysan Bio, Inc (Arab, AL, USA). Cyclo(Arg-Gly-Asp-D-Tyr-Lys) (RGD) peptide was purchased from Selleck Chemicals LLC (Houston, TX, USA). Cyclo(Arg-Ala-Asp-D-Phe-Lys-) (RAD) peptide was purchased from ANANSPEC (Fremont, CA, USA). (3-(4,5-dimethylthiazol-2-yl)-2,5-diphenyl tetrazolium bromide (MTT) was purchased from EMD Millipore corporation (Burlington, MA, USA).

1.2. Synthesis of HBGNCs

To create gold seeds (3 nm), 0.5 mL of 5 mM $HAuCl_4 \cdot 3H_2O$ and 0.5 mL of 5 mM Na_3Cit aqueous solutions were mixed with 9 mL of deionized water, followed by magnetic stirring for 10 minutes. To reduce gold ions to gold atoms, 1.5 mL of 1M $NaBH_4$ aqueous solution was rapidly added to this solution and stabilized for at least 6 hours[1]. To create HBGNCs, gold nanospheres (GNSs, 35 nm) were prepared via seed-mediated growth[2]. Then, 6 mL of 25 mM $HAuCl_4 \cdot 3H_2O$, 2.4 mL of 20 mM KI, 6 mL of 5wt% PVP, and 2.4 mL of 100 mM LAA aqueous solutions were added to 48 mL of deionized water, followed by magnetic stirring for 10 minutes. Under vigorous stirring, 1.8 mL of 3 nm-sized seeds were rapidly added, followed by stirring for 1 hour. The products (GNSs, 35 nm) were washed three times with deionized

water via centrifugation (10,000 rpm, 15 minutes), then dispersed in deionized water, adjusting the concentration to 3.32 nM.

HBGNCs were synthesized via surface-blocker assisted growth. Specifically, 0.1 mL of 10 wt% PVP and 0.25 mL of 5 mM $AgNO_3$ aqueous solutions were mixed with 4 mL of deionized water, followed by magnetic stirring for 5 minutes. Then, 0.2 mL of as-prepared GNSs (35 nm, 3.32 nM), 0.1 mL of 20 mM KI, and 25 mM $HauCl_4 \cdot 3H_2O$ aqueous solutions were rapidly added under magnetic stirring, followed by rapid addition of 0.5 mL of 200 mM LAA aqueous solution. To investigate the shape evolution of the HBGNCs, we controlled the volume of as-prepared GNSs (3.32 nM) from 0.2 to 1 mL in the growth process. In addition, to investigate the effect of the amount of surface blockers on the hyper-branch growth, we modulated the concentration of $AgNO_3$ and KI from 0.5 mM to 50 mM and from 2 mM to 200 mM in the growth step, respectively, while keeping the ratio of $AgNO_3$ to KI identical. After 30 minutes of the reaction, the products (HBGNCs) were washed three times with deionized water via centrifugation (9,000 rpm, 15 minutes) and then dispersed in deionized water for further use.

1.3. Characterization for HBGNCs

Transmission electron microscopy (HT 7700, Hitachi) and scanning electron microscopy (SU 8230 FE-SEM, Hitachi) were utilized to characterize the structure of HBGNCs. Extinction spectra of HBGNCs were analyzed by a UV-vis-NIR spectrophotometer (Evolution 220, Thermo Scientific). PA signals were analyzed by using the Vevo2100/LAZR imaging system (FujiFilm VisualSonics Inc.) with the LZ250 and LZ550 US transducers integrated with optical-fiber light delivery system for *in vitro* and *in vivo* studies, respectively. To measure PA signals from HBGNCs, GNRs, and GNSTs in polyethylene tube phantom, we prepared samples of different GNCs with the same solvent (water), the same optical density at peak optical absorption, and the same NC solution volume. Laser pulses were produced by a Q-switched

Nd:YAG-pumped optical parametric oscillator (OPO) laser (pulse duration: 7 ns, frame rate: 20 Hz). The gain in PA images and B-modes was set to 40 dB and 18 dB, respectively.

1.4. Numerical simulation

Optical cross-sections, including extinction, absorption, and scattering for GNSs, HBGNCs, GNRs, and GNSTs were calculated by a FDTD simulation (Lumerical Inc.). For our FDTD computational analysis, the surrounding temperature was 300 K. The optical characteristics of gold, such as dielectric constant and refractive index, were taken from the value in Johnson and Christy[3]. A total-field/scattered-field (TF/SF) source with a 500-900 nm wavelength range for the GNS and a 700-900 nm wavelength range for the HBGNC, GNR, and GNST was utilized to calculate their optical extinction, absorption, and scattering cross-sections. For our computational analysis, a single GNC was embedded in water because UV-vis-NIR spectra of different GNCs and their PA signals in our *in vitro* PA experiments were acquired in water (Figure S17). The structural parameters for the simulation model were based on TEM images of different GNCs. The optical extinction, absorption, and scattering cross-sections for different GNCs were calculated by taking an average value from the optical cross-sections under light excitation at different light polarization directions. Moreover, we set a maximum mesh step as 0.5 nm for the calculation of optical characteristics of different GNCs.

1.5. Surface functionalization of HBGNCs

To couple cyclic RGD peptides to HBGNCs, HBGNCs were first functionalized by CM-PEG-SH via gold-thiol chemistry. Specifically, 1 mL of HBGNCs (6.64 nM) was mixed with 9 mL of 1 mM CM-PEG-SH aqueous solution, followed by magnetic stirring for at least 12 hours and subsequently washing three times with deionized water via centrifugation (9,000 rpm, 15 minutes). The PEGylated HBGNCs were dispersed in 1 mL of deionized water and then mixed with 0.1 mL of 20 mM EDC and 20 mM NHS aqueous solutions, followed by shaking for 30

minutes. The EDC-NHS activated HBGNCs were washed twice with deionized water via centrifugation (9,000 rpm, 15 minutes) and subsequently dispersed in 1 mL of phosphate buffered saline (Corning, PBS) solution. 1 mL of 1 mM cyclic RGD aqueous solution was added to the EDC-NHS activated HBGNCs, followed by shaking for at least 12 hours. The RGD-coupled HBGNCs were washed three times with PBS solution via centrifugation (9,000 rpm, 15 minutes) and dispersed in 1 mL of PBS solution, adjusting the construct concentration as 6.64 nM.

1.6. Cell culture and cytotoxic test of HBGNCs

MDA-MB 231 breast cancer cells were cultured in Dulbecco's Modified Eagle's Medium (DMEM, Corning) containing 10% fetal bovine serum (Corning), 1 % penicillin-streptomycin by incubating at 37 ^{o}C in the presence of 5 % CO_2. For the cytotoxic test of HBGNCs, MDA-MB 231 cells were plated in a 96-well plate at a cell density of 8,000 cells per well. RGD-coupled HBGNCs with different concentrations (from 0 to 0.6 nM) were added to each well and incubated for 24 hours. After 24-hour incubation, free HBGNCs were removed and 0.2 mL of culture medium containing MTT (0.5 mg mL^{-1}) was added to each well, followed by incubation for 3 hours. The culture medium was removed and DMSO was then added to each well. The absorbance at 570 nm of the DMSO solution in each well was measured using a well plate reader (Synergy HY, BioTek).

1.7. *In vitro* PA cancer cell imaging

MDA-MB 231 cells were plated in a 6-well plate at a cell density of 100,000 cells per well. RGD-coupled HBGNCs were added to each well by adjusting the construct concentration of 0.075 nM. The HBGNCs were incubated with MDA-MB 231 cells for 24 hours to label the cells. To remove free HBGNCs, the cells were carefully rinsed with PBS solution three times and then collected via trypsinization. Labeled cells were resuspended in separate tubes at a

volume of 20 µL at various concentrations from 38 cells µL^{-1}to 1,200 cells µL^{-1}. The labeled cell samples were mixed with 20 µL of 16 % gelatin solution as a precursor and then pipetted to create a dome onto a gelatin/silica-based tissue-mimicking phantom base. To measure PA signals and contrast from each dome, the phantom base was placed in the Vevo2100/LAZR imaging system, followed by US/PA imaging at 700 nm. The PA gain and B-mode gain was set as 40 dB and 18 dB, respectively.

1.8. *In vivo* PA cancer imaging

All *in vivo* experiments were carried out under the Institutional Animal Care and Use Committee (IACUC) protocols of the Georgia Institute of Technology. To investigate PA responses of HBGNCs *in vivo*, HBGNCs (50 µL, 1.2 nM in PBS solution) were mixed with Matrigel (Corning, 50 µL). The mixture was subcutaneously injected into mice (The Jackson Laboratory, 6-8 weeks old, female, NU/J), followed by imaging in the Vevo2100/LAZR system. The HBGNCs were imaged within the 700-900 nm spectral range. The PA gain and B-mode gain was set as 40 dB and 18 dB, respectively.

For *in vivo* PA cancer imaging, MDA-MB 231 cells (50 µL) at a cell concentration of 20,000 cells µL^{-1} in 50% Matrigel were subcutaneously inoculated into the flank of mice (The Jackson Laboratory, 6-8 weeks old, female, NU/J). After tumor volumes reached over 100 mm^3, 60-80 µL of RGD-coupled HBGNCs in PBS solution (1.6 nM) was intravenously injected via the tail-vein injection. The saline solution (60-80 µL) was used as a control group. After 24 hours of the injection of the HBGNC or saline solution, the mice were anesthetized and then placed on a heating plate in the Vevo2100/LAZR system. US/PA imaging for the tumor region was performed within the 700-900 nm spectral range. The PA gain and B-mode gain was set as 40 dB and 18 dB, respectively. All the *in vivo* data were post-processed in MATLAB (MathWorks, Inc.).

Supplementary Note 2. PA signal generation from different GNCs

1. Calculation of the optical absorption coefficients of GNCs with different geometries

In PA imaging, PA signal amplitude of GNCs in the water is estimated as follows[4–6]:

$$p_0 = \frac{\beta c^2}{C_p}\mu_a F = \Gamma \mu_a F \quad (1)$$

where p_0 is the photoacoustic pressure, β is the thermal expansion coefficient of the water, c is the sound speed in the water, C_p is the specific heat capacity of the water, μ_a is the optical absorption coefficient of GNCs in the water, F is the laser fluence, and Γ is the Grüneisen parameter, respectively.

When laser fluence and optical density are kept constant for PA experiment of each GNC, the only variable to change PA signal generation from the GNC aqueous solutions is the fraction of the optical absorption coefficient and the extinction coefficient, i.e., absorption efficiency, of the GNCs. It should be noted that the optical density of GNCs is associated with their optical extinction coefficient (μ_e), not absorption coefficient.

$$\mu_e = \mu_a + \mu_s \quad (2)$$

$$p_0 = \frac{\beta c^2}{C_p}\mu_a F = \Gamma \mu_a F \propto \mu_a \quad (3)$$

The absorption efficiency for each GNC is calculated as follows:

$$\frac{\mu_a}{\mu_e} = \frac{\sigma_{absorption} C}{\sigma_{extinction} C} = \frac{\sigma_{absorption}}{\sigma_{extinction}} \quad (4)$$

where $\sigma_{absorption}$ and $\sigma_{extinction}$ are absorption and extinction cross-sections for each GNC and C is the concentration of each GNC, respectively. The absorption and extinction cross-sections for each GNC can be calculated via an FDTD simulation.

Supplementary Note 3. Supplementary Figures

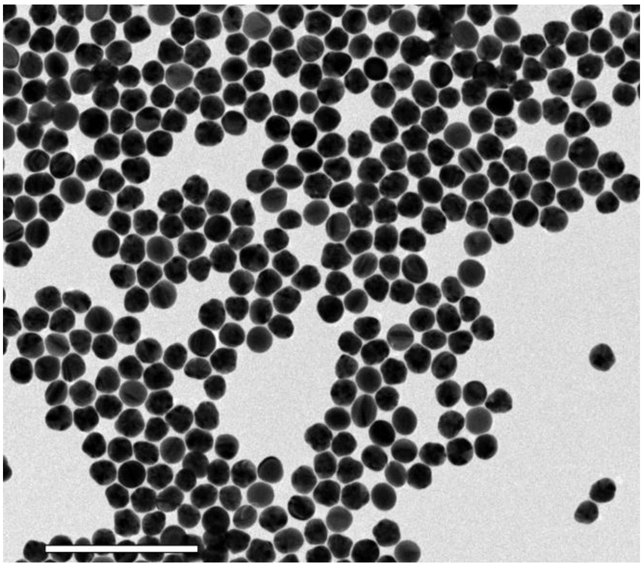

Figure S1 | A low-magnification TEM image of GNSs (35 nm) as a seed particle for hyper-branch growth. The scale bar is 200 nm.

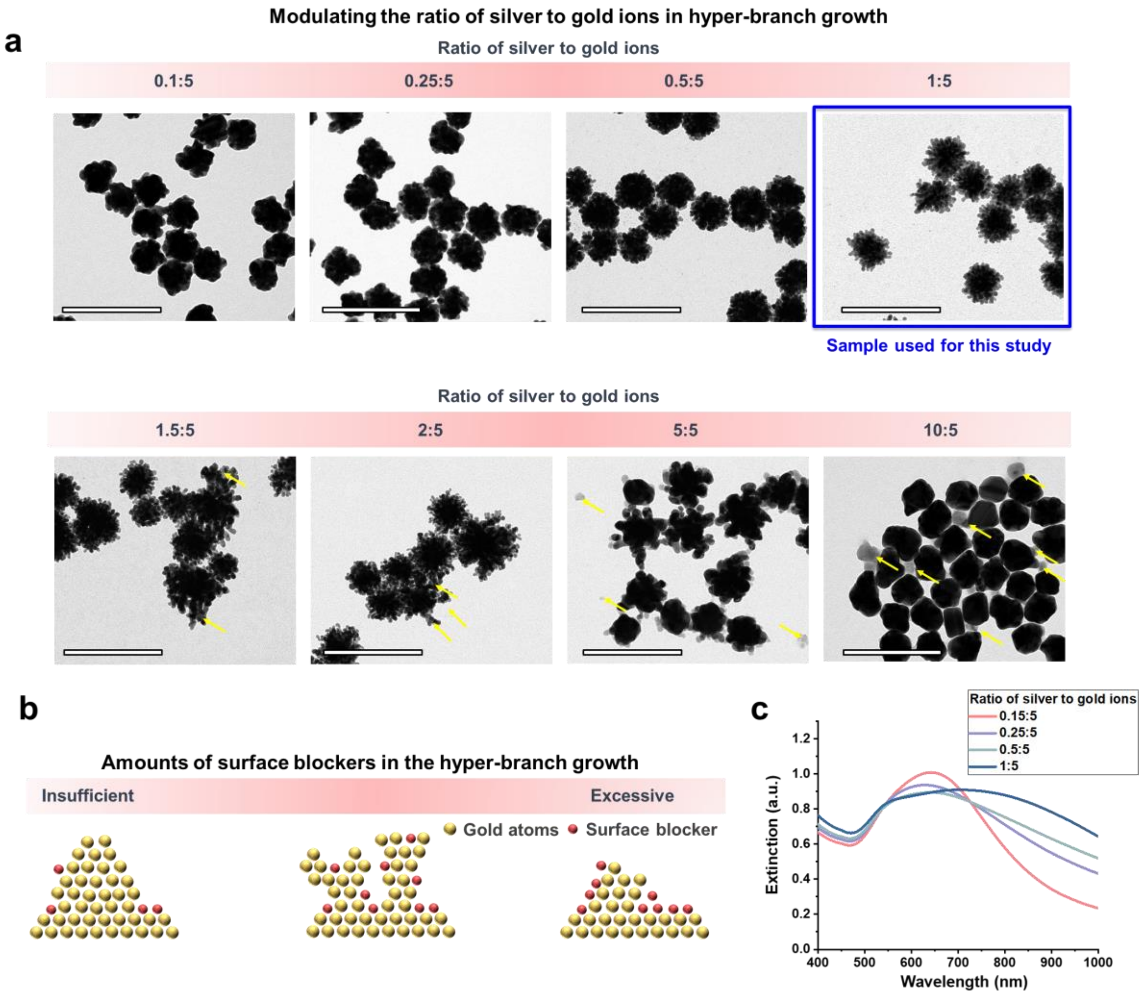

Figure S2 | a) TEM images of GNCs synthesized at different ratios of silver to gold ions in the seed-mediated growth of GNSs. In this experiment, we maintained the silver-to-halide ion ratio and the total amount of gold ions constant to investigate the effect of the blocked surface density of GNSs by the silver-halide complexes on the hyper-branch growth. The yellow arrows indicate free nucleated particles. Scale bars are 200 nm. b) A schematic depicting the effect of the surface blocker quantity on hyper-branch growth. c) UV-vis-NIR spectra of HBGNCs synthesized at silver-to-gold ion ratios ranging from 0.1:5 to 1:5. The particle concentration for each HBGNC was adjusted at 26 pM.

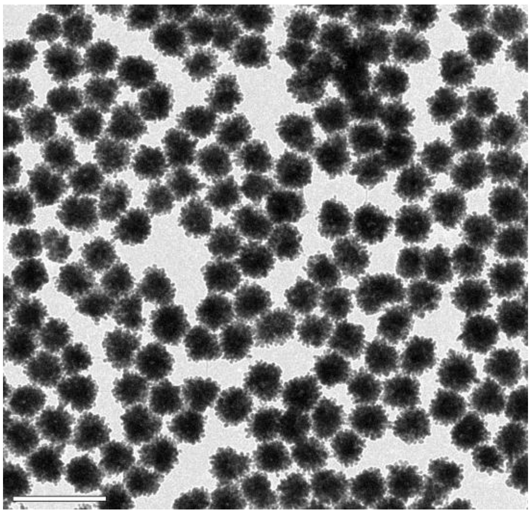

Figure S3 | A low-magnification TEM image of HBGNCs. The scale bar is 200 nm.

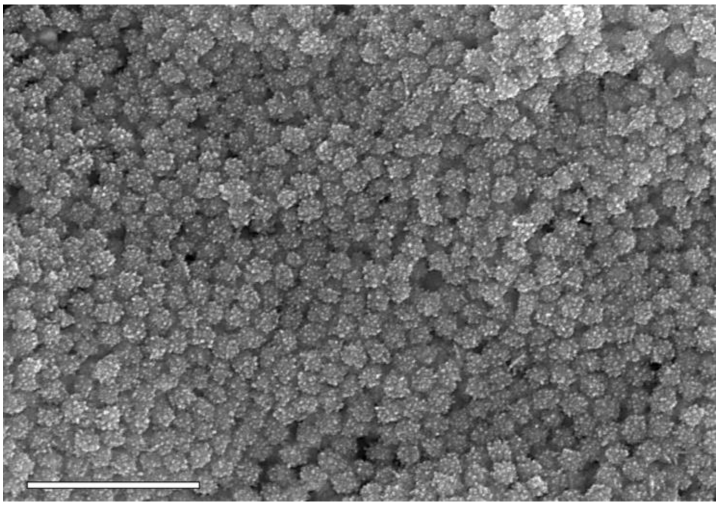

Figure S4 | A low-magnification SEM image of HBGNCs. The scale bar is 500 nm.

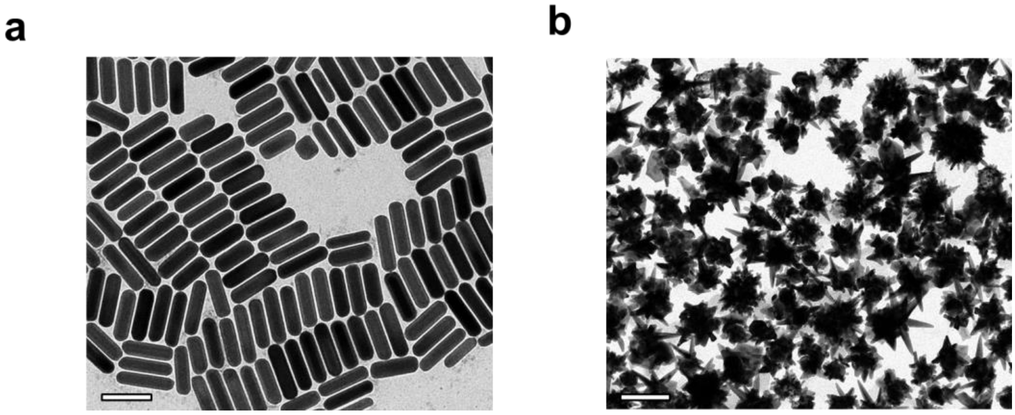

Figure S5 | (a, b) Low-magnification TEM images of GNRs and GNSTs. The scale bars are 100 nm. The GNRs and GNSTs were synthesized as previously reported seed-mediated growth methods[7,8].

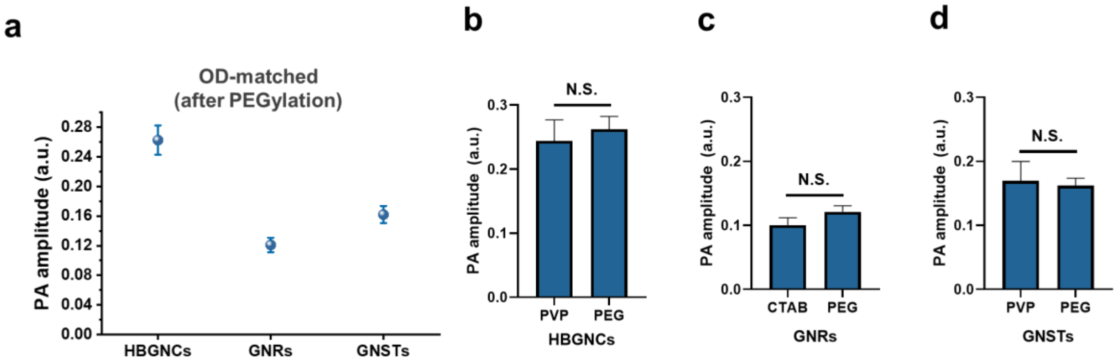

Figure S6 | (a) PA signal generation from OD-matched GNCs after PEGylation. PA signal amplitude from the HBGNCs, GNRs, and GNSTs were acquired at 700 nm, 830 nm, and 750 nm, respectively (n=5). (b-d) Comparison of PA amplitude of different GNCs before and after the PEGylation process (n=5). Data are presented as the mean ± standard deviation. The statistical analysis for Figures S6b-d was conducted using Student's t-test. N.S. indicates no significant differences.

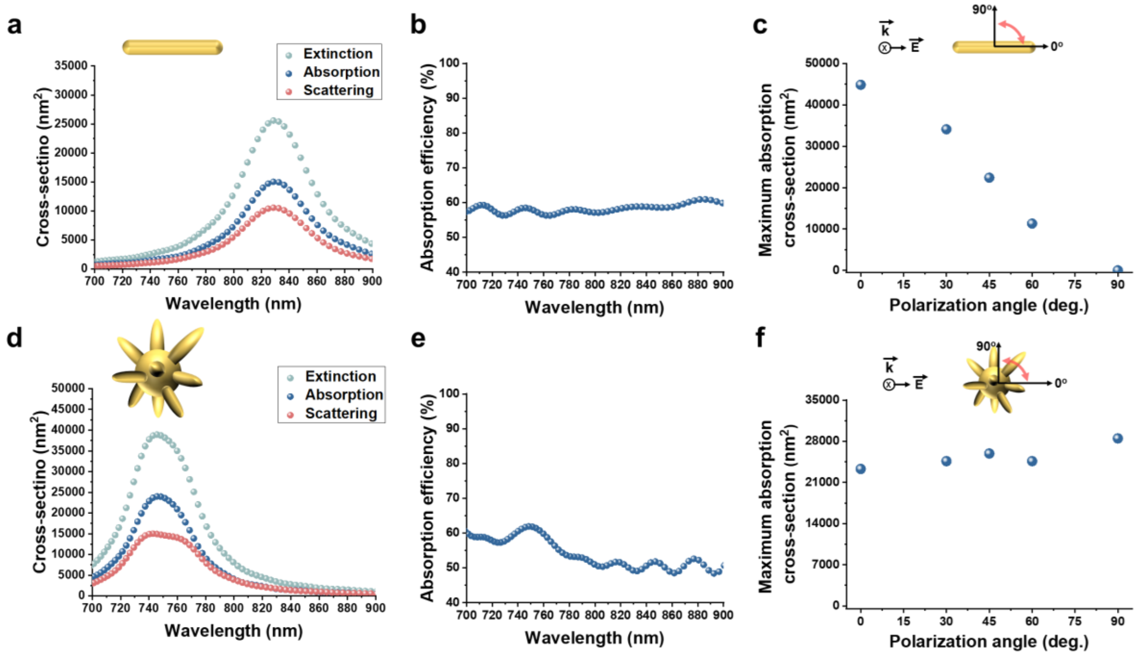

Figure S7 | (a-f) Calculated extinction, absorption, and scattering cross-sections, absorption efficiencies, and maximum absorption cross-section at different light polarization directions for GNR and GNST within the 700-900 nm spectral range.

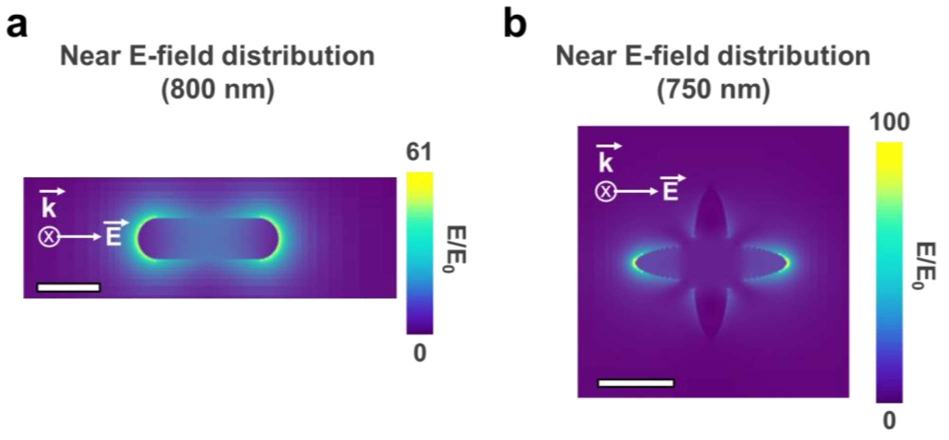

Figure S8 | (a, b) Calculated near E-field distributions of GNR and GNST at the peak wavelength of optical absorption. The scale bars are 40 nm.

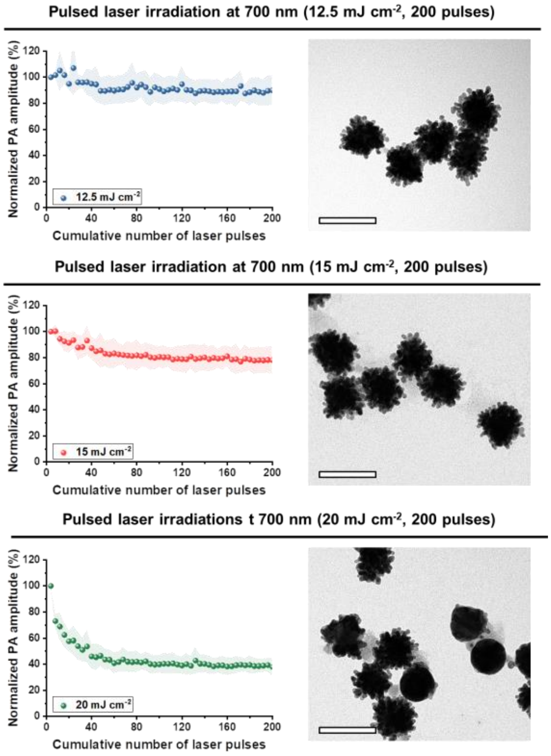

Figure S9 | Measurement of PA signal generation from HBGNCs for 200 laser pulses (700 nm wavelength) at different laser fluences, including 12.5 mJ cm^{-2}, 15 mJ cm^{-2}, and 20 mJ cm^{-2} (n=5), and TEM characterization for structural stability of HBGNCs after pulsed laser illumination at the corresponding laser fluences. Data are presented as the mean ± standard deviation (n=5). Scale bars are 100 nm.

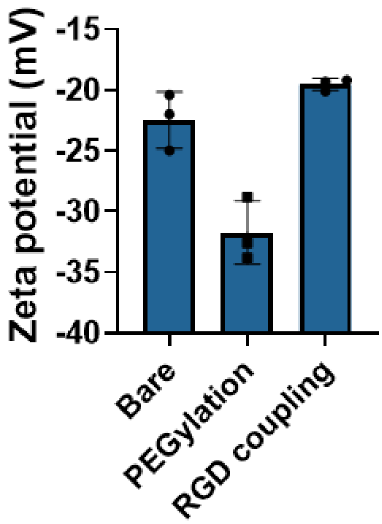

Figure S10 | Zeta potential analysis to investigate the serial surface chemistry for coupling cyclic RGD ligands to the surface of HBGNCs (n=3). Data are presented as the mean ± standard deviation.

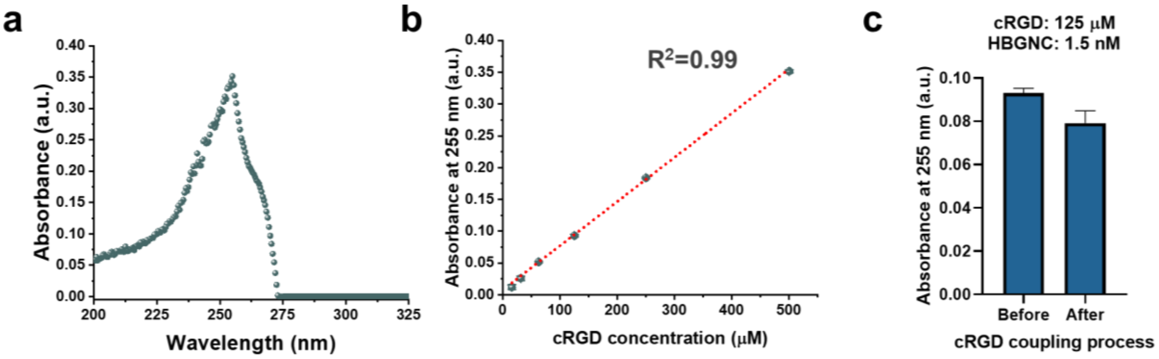

Figure S11 | a) UV-vis spectrum of cRGD solutions at the concentration of 500 μM. b) Absorbance (255 nm) of cRGD solutions at different cRGD concentrations (n=3). c) The difference of the absorbance of cRGD solutions at 255 nm before and after cRGD coupling process (n=3). Data are presented as the mean ± standard deviation.

The quantification of cRGD ligands per HBGNC was performed using UV-vis spectroscopy, following a previously reported method[9]. Naïve cRGD ligands exhibited strong absorbance at 255 nm, as shown in Figure S11a. The absorbance of cRGD solution demonstrated a linear increase with increasing RGD concentration (Figure S11b). After a 24-hour incubation of cRGD ligands with EDC/NHS-activated HBGNCs, the mixture was centrifuged, and the supernatant was collected. The absorbance spectrum of supernatant was measured and directly compared with cRGD solutions at a known concentration (125 μM) to quantify the density of cRGD ligands per a single HBGNC. By calculating the difference in absorbance between the cRGD solutions before and after the cRGD coupling process, we determined the density of cRGD ligands on HBGNCs to be 6,278 ± 1,456 ligands per HBGNC (Figure S11c).

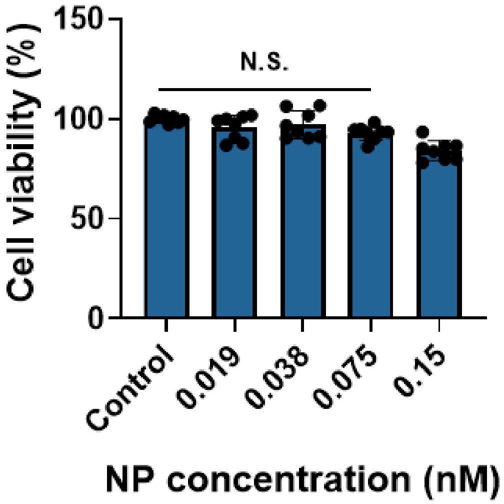

Figure S12 | Cell viability of MDA-MB 231 cells after 24 h-incubation with cRGD-HBGNCs at varied construct concentrations (n=8). Data are presented as the mean ± standard deviation.

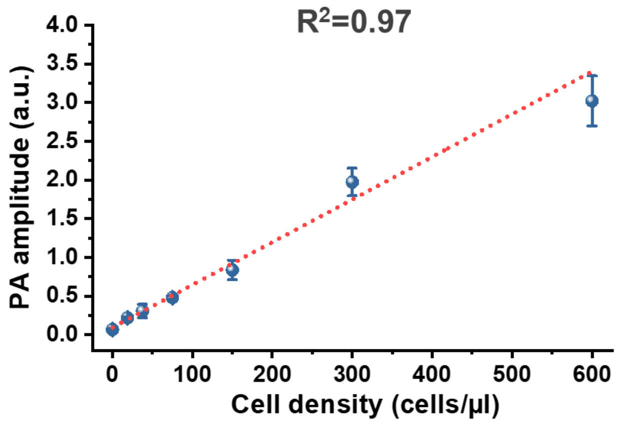

Figure S13 | Quantitative analysis for PA signal generation from MDA-MB 231 cells labeled with cRGD-HBGNCs at different cell densities (n=3). Data are presented as the mean ± standard deviation.

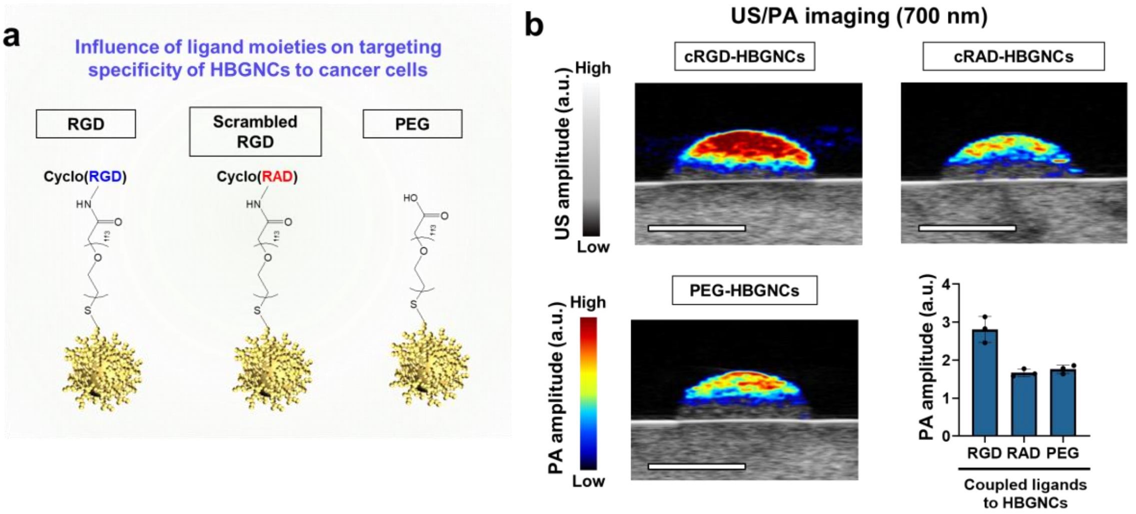

Figure S14 | a) Schematic of HBGNCs with different ligand moieties, such as cyclic RGD peptides, cyclic RAD peptides (scrambled RGD sequence), and PEG ligands. b) US/PA images (700 nm wavelength) of the dome phantoms containing labeled MDA-MB 231 cells at the cell density of 600 cells μl^{-1} under pulsed laser illumination and the corresponding quantification of PA amplitudes for each dome phantom. The scale bars are 4 mm. Data are presented as the mean ± standard deviation. The imaging experiments were repeated independently three times and similar results were obtained.

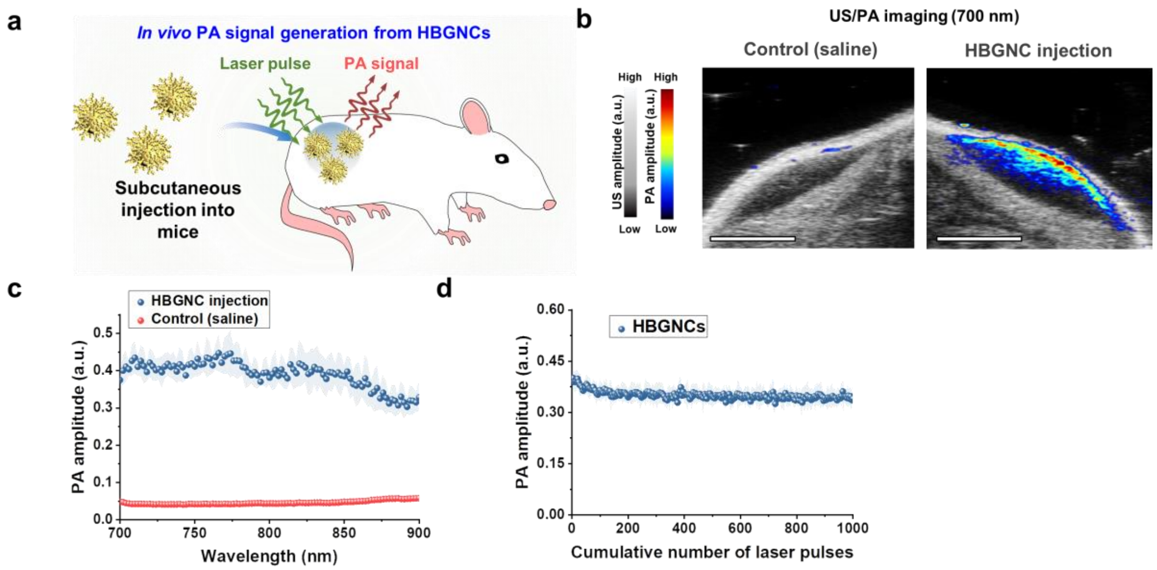

Figure S15 | (a) Schematic depicting *In vivo* US/PA imaging of the subcutaneously injected HBGNCs. (b) US/PA images of saline and HBGNC solutions in 50% Matrigel under pulsed laser illumination at 700 nm. (c) PA spectra from the saline and HBGNC solutions within the 700-900 nm spectral range (n=3). (d) *In vivo* PA signals from HBGNCs under 700 nm-pulsed laser illumination for 1,000 pulses at 10 mJ cm^{-2} (n=3). Data are presented as the mean ± standard deviation. The imaging experiments were repeated independently three times and similar results were obtained.

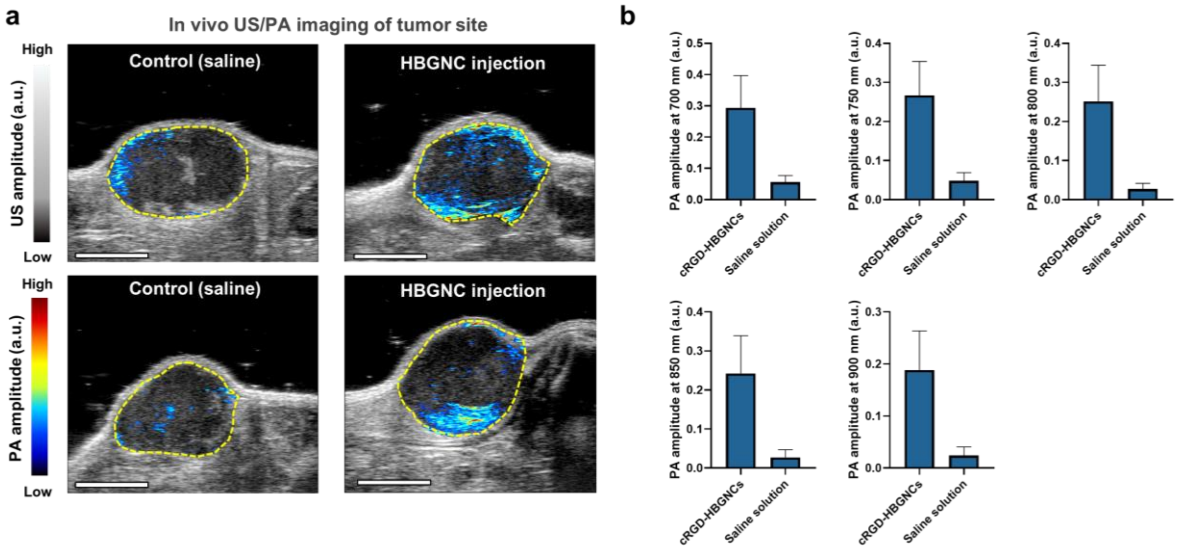

Figure S16 | a) US/PA images (700 nm wavelength) of the tumor region with or without the intravenous injection of the cRGD-HBGNCs to mice. The scale bars are 4 mm. The yellow dotted contour indicates the tumor region based on US imaging. This US/PA imaging result shows that our in vivo tumor imaging experiments for Figure 4e were repeated independently three times and similar results were received. b) Quantification of the difference in PA signal amplitude between the tumor sites that received cRGD-HBGNCs or saline solutions (n=3). Data are presented as the mean ± standard deviation.

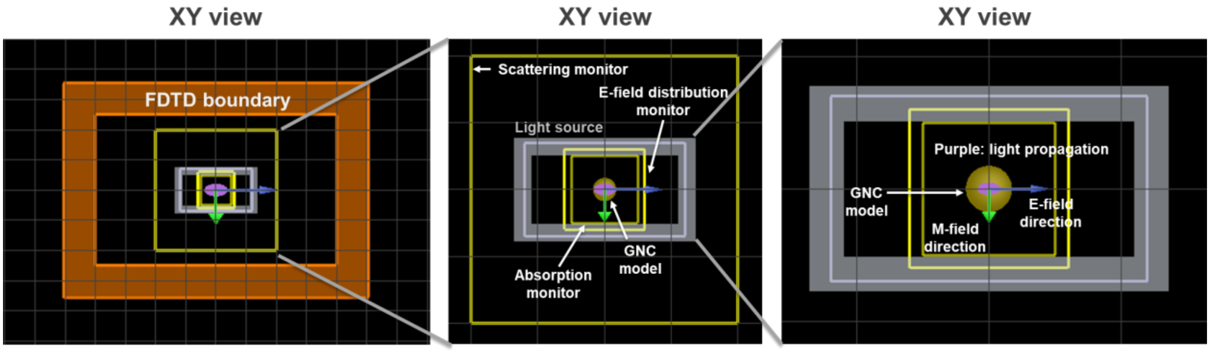

Figure S17 | Schematics for the FDTD simulation model of GNCs in water based on the view of XY plane.

References

(1) Kim, J.; Seo, D.; Lee, J.; Southard, K. M.; Lim, Y.; Kim, D.; Gartner, Z. J.; Jun, Y.; Cheon, J. Single-Cell Mechanogenetics Using Monovalent Magnetoplasmonic Nanoparticles. *Nat. Protoc.* **2017**, *12* (9), 1871–1889. https://doi.org/10.1038/nprot.2017.071.

(2) Gao, C.; Vuong, J.; Zhang, Q.; Liu, Y.; Yin, Y. One-Step Seeded Growth of Au Nanoparticles with Widely Tunable Sizes. *Nanoscale* **2012**, *4* (9), 2875. https://doi.org/10.1039/c2nr30300k.

(3) Johnson, P. B.; Christy, R. W. Optical Constants of the Noble Metals. *Phys. Rev. B* **1972**, *6* (12), 4370–4379. https://doi.org/10.1103/PhysRevB.6.4370.

(4) Cox, B. T.; Beard, P. C. Fast Calculation of Pulsed Photoacoustic Fields in Fluids Using *k* -Space Methods. *J. Acoust. Soc. Am.* **2005**, *117* (6), 3616–3627. https://doi.org/10.1121/1.1920227.

(5) Chen, Y.-S.; Frey, W.; Aglyamov, S.; Emelianov, S. Environment-Dependent Generation of Photoacoustic Waves from Plasmonic Nanoparticles. *Small* **2012**, *8* (1), 47–52. https://doi.org/10.1002/smll.201101140.

(6) Beard, P. Biomedical Photoacoustic Imaging. *Interface Focus* **2011**, *1* (4), 602–631. https://doi.org/10.1098/rsfs.2011.0028.

(7) Ye, X.; Zheng, C.; Chen, J.; Gao, Y.; Murray, C. B. Using Binary Surfactant Mixtures To Simultaneously Improve the Dimensional Tunability and Monodispersity in the Seeded Growth of Gold Nanorods. *Nano Lett.* **2013**, *13* (2), 765–771. https://doi.org/10.1021/nl304478h.

(8) Yuan, H.; Khoury, C. G.; Hwang, H.; Wilson, C. M.; Grant, G. A.; Vo-Dinh, T. Gold Nanostars: Surfactant-Free Synthesis, 3D Modelling, and Two-Photon Photoluminescence Imaging. *Nanotechnology* **2012**, *23* (7), 075102. https://doi.org/10.1088/0957-4484/23/7/075102.

(9) Kim, Y.-H.; Jeon, J.; Hong, S. H.; Rhim, W.-K.; Lee, Y.-S.; Youn, H.; Chung, J.-K.; Lee, M. C.; Lee, D. S.; Kang, K. W.; Nam, J.-M. Tumor Targeting and Imaging Using Cyclic RGD-PEGylated Gold Nanoparticle Probes with Directly Conjugated Iodine-125. *Small* **2011**, *7* (14), 2052–2060. https://doi.org/10.1002/smll.201100927.
